# Current Insights into Superinfection Exclusion in Insect-Specific Orthoflaviviruses

**DOI:** 10.3390/v18010115

**Published:** 2026-01-15

**Authors:** Justin J. X. Chan, Ziyao Zhao, Carla J. S. P. Vieira, Jarvis Z. H. Goh, Andrii Slonchak

**Affiliations:** 1 Systems Virology Laboratory, QIMR Berghofer Medical Research Institute, Brisbane, QLD 4006, Australia; 2 School of Chemistry and Molecular Biosciences, The University of Queensland, Brisbane, QLD 4072, Australia; 3 Australian Infectious Diseases Research Centre, Global Virus Network, Brisbane, QLD 4072, Australia

**Keywords:** *Orthoflavivirus*, superinfection exclusion, insect-specific flaviviruses, biocontrol

## Abstract

The *Orthoflavivirus* genus includes a variety of human-pathogenic, mosquito-borne flaviviruses (MBFs) including dengue, Zika, and West Nile viruses, which pose significant global public health threats. Insect-specific flaviviruses (ISFs) are another group within the genus that exclusively replicate in mosquitoes and are incapable of infecting vertebrates. ISFs have recently attracted growing research interest due to their potential applications in vaccine development. In addition, multiple studies have demonstrated that prior infection with ISFs such as Palm Creek virus and Binjari virus can suppress subsequent infection with human-pathogenic MBFs. This phenomenon, known as superinfection exclusion (SIE), opens the avenue for the potential applications of ISFs in MBF transmission control. This prompted a growing number of studies into ISFs and their interactions with MBFs in mosquito hosts. In this review, we provide an overview on ISFs, with a particular emphasis on the capacity of different ISFs to cause SIE, the current insights into the mechanisms of this phenomenon, and the potential use of ISFs in the SIE-based biocontrol strategies.

## 1. Introduction

**Figure 1:**
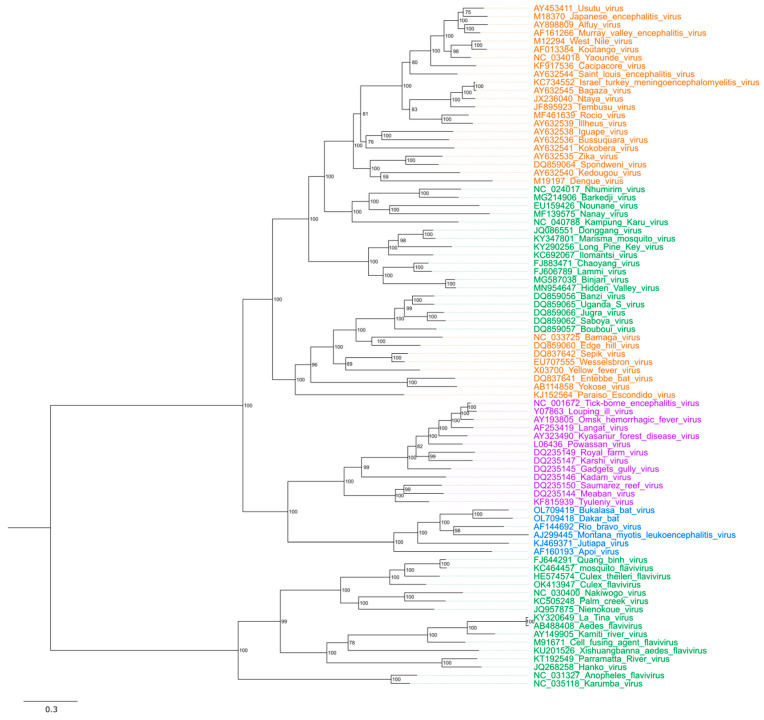
Phylogenetic tree of *Orthoflavivirus* genus. Clades are coloured according to their ecological groups: mosquito-borne (MBFs) in orange, tick-borne (TBFs) in purple, no known vector (NKVFs) in blue, and insect-specific flaviviruses (ISFs) in green. Viral genomes were selected based on the previous studies [12,13,14] and obtained from GenBank using the indicated sequence accession numbers. ModelFinder was used to select the best evolutionary model [15]. Consensus maximum-likelihood trees were constructed with IQ-TREE v3.0.1 [16] using the LG + F + R8 amino acid substitution model and 1000 Ultrafast bootstraps [17] with bootstrap support labelled on each node. Tree is midpoint rooted. Branch length represents the number of amino acid substitutions per site. Tree visualisation and figure generation were performed with FigTree v1.4.4 [18].

The family of *Flaviviridae* contains four genera: *Pegivirus*, *Hepacivirus*, *Pestivirus*, and *Orthoflavivirus*. All *Flaviviridae* members are enveloped viruses that consist of a protein-encapsulated, positive-sense (+), single-stranded (ss) RNA ranging 9–13 kb in length [1]. The *Orthoflavivirus* genus consists of approximately 70 viral species that primarily infect mammals, birds, and insects [2]. According to the transmission vector and host range, the *Orthoflavivirus* genus is classified into four ecological groups: mosquito-borne (MBFs), tick-borne (TBFs), no known vector (NKVFs), and insect-specific flaviviruses (ISFs) [3] (Figure 1). MBFs and TBFs, collectively known as dual-host orthoflaviviruses, constitute the largest group within the genus and pose a major public health concern due to the prevalence of numerous human pathogens in this category [1,2]. Examples of pathogenic MBFs include Zika virus (ZIKV), dengue virus (DENV), Japanese encephalitis virus (JEV), yellow fever virus (YFV), and West Nile virus (WNV).

MBFs are primarily maintained in sylvatic transmission cycles, with spillovers into humans leading to outbreaks for viruses such as WNV, JEV or SLEV with humans being dead-end hosts [4,5]. Urban human–mosquito–human cycles are known to occur for viruses such as DENV and YFV [5]. In the sylvatic cycle, mosquitoes become infected by feeding on infected animal hosts, allowing the virus to replicate and spread within the mosquito [6]. The infected mosquito then transmits the virus to new hosts through saliva during subsequent bites [7]. Humans are typically not involved in the sylvatic cycle as it occurs in forest areas. However, changes in climate and human activities may introduce humans as hosts, initiating the urban cycle [8,9,10]. The primary vectors of MBFs are mosquitoes from the genera *Aedes* (e.g., *Ae. aegypti* and *Ae. albopictus*), *Culex* (e.g., *Cx. pipiens*), and *Haemagogus* (e.g., *Hg. janthinomys* and *Hg. equinus*) [11], while the non-human hosts include mammals and birds [10].

Currently, there is no specific treatment for pathogenic orthoflaviviruses. Besides avoiding exposure to the arthropods, vaccination is another protective strategy that can reduce the spread of the pathogenic orthoflaviviruses. Licenced vaccines are currently available for several MBFs. These include the live-attenuated yellow fever vaccine YF-VAX [19], the inactivated JEV vaccine JEspect (Ixiaro), and the live-attenuated JEV vaccine Imojev, all of which have demonstrated high efficacy and safety and are widely used globally [20]. However, many vaccines have administration restrictions. For example, the dengue vaccine CYD-TDV (Dengvaxia) is only recommended for individuals previously infected with DENV [21]. Currently, no human vaccines are commercially available for ZIKV and WNV.

Insect-specific orthoflaviviruses are a large group of viruses within the *Orthoflavivirus* genus that replicate exclusively in insect hosts [22]. Since they are incapable of replicating in vertebrate cells, ISFs pose no risk to human health. This makes them attractive platforms for the recombinant vaccine design [23]. In addition, some ISFs are considered as potential tools for biocontrol of vertebrate-infecting orthoflaviviruses [24] due to their ability to supress replication of the pathogenic viruses in mosquitoes, in a phenomenon known as superinfection exclusion. Therefore, ISFs, once largely overlooked, are now gaining increasing research attention.

Superinfection exclusion, also known as viral interference, is a phenomenon where a pre-existing (primary) viral infection within a host inhibits or significantly reduces subsequent (secondary) viral infection [25,26]. This phenomenon has been observed across various viral families [25,26,27,28,29,30,31,32] and can be categorised into three types of viral interferences: homologous, heterologous, and heterotypic [33]. Homologous viral interference occurs when both the primary and secondary viruses are from the same family [34]. In contrast, when the viruses belong to different families, it is termed heterologous viral interference [35]. A specific form of homologous viral interference is known as heterotypic viral interference, where both the primary and secondary viruses belong to the same species but are of different serotypes [36]. In some cases, two different viruses can co-exist within the same host without interfering with each other; this phenomenon is termed as viral accommodation [37]. Interestingly, ISFs have been shown to interact with human-pathogenic orthoflaviviruses in insect vectors and alter vector competence in both positive [38,39] and negative ways [39,40], which highlights the need for deeper understanding of the underlying mechanisms of superinfection exclusion (SIE) and ISF–host interactions prior to utilisation of ISFs in biocontrol applications in the field.

This review focuses on homologous SIE in ISFs, providing a comprehensive and updated summary of current knowledge (Table 1, Table S1) alongside an updated *Orthoflavivirus* phylogenetic tree that incorporates recently identified MBFs and ISFs. It also discusses the most recent insights into the mechanisms underlying SIE, as well as the potential utilisation of ISFs in *Orthoflavivirus* biocontrol strategies.

## 2. Molecular Organisation and Replication of Orthoflaviviruses

**Figure 2:**
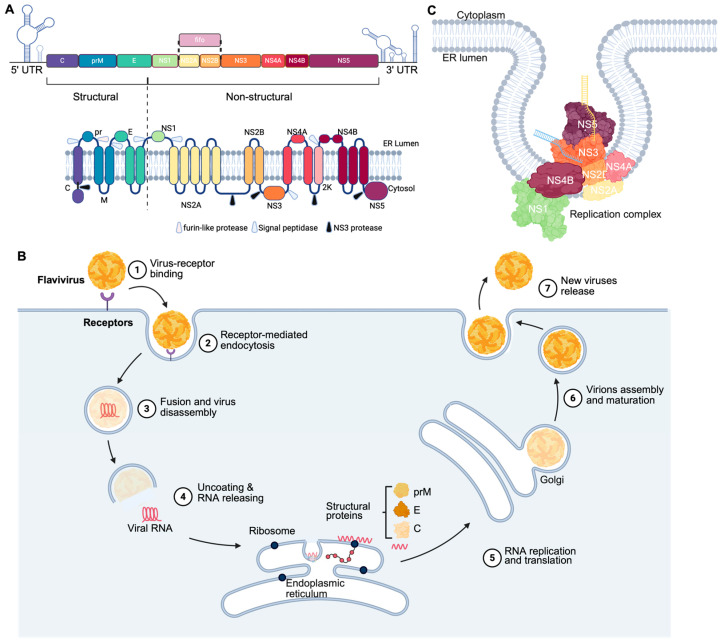
*Orthoflavivirus* genome organisation and replication. (**A**) *Orthoflavivirus* genome organisation. The viral genome consists of an open reading frame (ORF) flanked with 5′UTR and 3′UTR. The ORF is firstly translated into a polyprotein which is co- and post-translationally cleaved by various proteases (furin-like, signal and NS3 protease) to generate three structural proteins (C, prM, and E) and seven non-structural proteins (NS1, NS2A, NS2B, NS3, NS4A, NS4B, and NS5); the fifo gene overlaps the NS2A/NS2B region. (**B**) *Orthoflavivirus* replication process. Infection of the susceptible cells begins with the receptor binding followed by receptor-mediated endocytosis. After membrane fusion, the viral RNA is released and translated into a polyprotein within the endoplasmic reticulum (ER). The polyprotein is then cleaved into three structural and seven NS proteins. The NS proteins form the replication complex. The RNA functions as the template, and new RNAs are synthesised within the complex. The newly synthesised RNAs are enclosed with structural proteins in the ER, transported to the Golgi apparatus, whereby the viral particles are formed and matured, and subsequently released through exocytosis. (**C**) *Orthoflavivirus* RNA replication complex. The replication complex forms on ER membranes and consists of seven NS proteins that remodel ER membranes and support viral RNA synthesis. Created in BioRender. Zhao, Z. (2026). https://BioRender.com/fngft2l.

All orthoflaviviruses, including ISFs, possess a positive-sense, single-stranded RNA genome encapsidated in structural proteins and a lipid envelope. Their genomes (Figure 2A) are approximately 11 kb in length and include a type I (m7GpppAm) cap at the 5′ end, while lacking a poly(A) tail at the 3′ end [22]. The large open reading frame (ORF) of orthoflaviviruses is flanked by 5′ and 3′ untranslated regions (UTRs) that are roughly 100 nucleotides (nt) and 400 to 700 nt in length, respectively [41]. The UTRs contain essential elements for translation and replication of viral RNA, such as 5′ and 3′ upstream AUG regions (UAR) and 5′ and 3′ cyclisation sequences (5′CS and 3′CS) [42,43]. In addition, *Orthoflavivirus* 3′UTRs encode viral noncoding RNAs, known as subgenomic flaviviral RNAs (sfRNAs) [44]. The sfRNAs are produced by all known orthoflaviviruses including dual-host viruses, ISFs, and NKVFs [13,45,46,47] and act as suppressors of the host antiviral responses in mosquito [48] and vertebrate [44,49,50] hosts.

Viral ORF encodes for a single polyprotein, which is cleaved by viral and host proteases into seven non-structural (NS1, NS2A, NS2B, NS3, NS4A, NS4B, and NS5) and three structural (capsid (C), pre-membrane (prM), and envelope (E)) proteins [51]. Most cleavage occurs co-translationally, with the exception of prM/E cleavage which is post-translational and results in furin-mediated processing of prM into the mature membrane (M) protein [52]. Additionally, NS2A/NS2B region of certain ISFs [53], including Culex flavivirus (CxFV), Quang Binh virus (QBV) [53], and Niénokoué virus (NIEV) contain a unique overlapping gene named fifo (fairly interesting flavivirus ORF) [54]. This gene overlaps the NS2A/NS2B coding region in the −1/+2 reading frame and is thought to encode for a trans-frame fusion protein via programmed ribosomal frameshifting [53].

*Orthoflavivirus* infection begins with cell entry, which relies on the binding of the E proteins to receptors on the cell surface. Entry receptors have been identified for DENV, JEV, and tick-borne encephalitis virus (TBEV) and include glycosaminoglycans (GAGs), C-type lectin receptors, and mannose receptors [55,56,57]. However, most of these receptors have been only characterised in mammalian cells. Only recently, heat shock proteins and laminin receptors have been identified as the entry receptors for JEV in the mosquito C6/36 cell line [57]. The specific entry receptors for ISFs are currently unknown.

The entire viral replication process includes seven steps (Figure 2B). After the E protein binds to the targeted receptor, the virus enters host cells via clathrin-mediated endocytosis, resulting in the fusion of the internalised viral particle membrane and the endosome membrane, accompanied by a reduction in intra-endosomal pH [56,58,59]. The decreasing pH environment promotes conformational changes in the E protein, which are essential for its insertion into the endosomal membrane and result in the formation of the fusion pore [56]. The nucleocapsid is then released into the cytosol through the enlarged pore. Subsequently, the viral capsid disassembles and the genomic RNA is released into the cytoplasm, which is then translated into the viral polyprotein on the endoplasmic reticulum (ER)-associated ribosomes [58]. The translated polyprotein is cleaved by the viral and host proteases, producing structural and non-structural viral proteins.

Replication of *Orthoflavivirus* RNA and assembly of virions occur within the replication compartments formed by the ER membrane (Figure 2C). NS proteins facilitate the rearrangement of the host ER membrane to create these compartments [60]. On the membranes of the replication compartment, viral NS proteins form the replication complex (RC) in which viral RNA is synthesised through the RNA-dependent RNA polymerase (RdRp) activity of NS5. The RC consists of NS2A, NS2B, NS4A, and NS4B anchored to the ER membrane, while NS1, NS3, and NS5 are recruited through protein–protein or protein–RNA interactions [61,62].

As replication of the viral genome progresses, the C protein binds to the newly synthesised RNA to form the nucleocapsid core [63,64]. The nucleocapsid then buds into the lumen of the ER, acquiring a lipid envelope embedded with prM and E proteins. The prM-E heterodimers of immature viral particles form rough protrusions on the surface and interact with each other to form an icosahedral lattice structure [64,65,66]. Parts of the prM cover the fusion peptide of the E protein, preventing premature fusion. In addition, prM helps maintain the proper folding of the E protein [64,65,67]. The assembled immature virions are transported to the Golgi apparatus for maturation. This involves structural rearrangement of prM and E proteins induced by acidic pH. As a result, the E proteins shift from trimeric spikes to flat dimers, a transition crucial for viral infectivity. In addition, the prM undergoes cleavage at its N-terminal region by the host protease furin, resulting in the formation of the pr peptide and the M protein [64,65]. The pr peptide remains transiently associated with the virion, preventing premature membrane fusion [68,69]. Finally, the new mature virions are released by exocytosis, during which the pr fragment dissociates, yielding a mature infectious virion [69,70].

Overall, the molecular mechanisms of *Orthoflavivirus* replication and functions of individual viral proteins are currently well characterised (reviewed in [59,61,71,72]). However, most information to date is based on human-pathogenic MBFs, while molecular biology of ISFs is not clearly defined, and the functions of ISF proteins in virus–host interactions have not been specifically addressed.

## 3. Diversity of the Insect-Specific Orthoflaviviruses

Insect-specific orthoflaviviruses constitute a distinct group within the *Orthoflavivirus* genus, defined by their ability to infect only insect cells and inability to replicate in vertebrates [73,74]. ISFs can be divided into two major groups: classical insect-specific flaviviruses (cISFs) and dual-host-associated insect-specific flaviviruses (dISFs). The cISFs evolved separately from the ancient *Orthoflavivirus* progenitor and represent a distinct clade from the vertebrate-infecting viruses. [14] The dISFs are assumed to have evolved from dual-host orthoflaviviruses that lost the ability to infect vertebrate cells [13,22,75]. They phylogenetically cluster together with mosquito-borne dual-host orthoflaviviruses (Figure 1). These evolutionary patterns highlight the distinct host adaptations and divergence that have shaped the diversification of ISFs within the *Orthoflavivirus* genus.

### 3.1. Lineage I or Classical Insect-Specific Flaviviruses

The discovery of cISF dates back to 1975, when cell fusing agent virus (CFAV) was first isolated from *Ae. aegypti* cell cultures [76]. Initially, CFAV received little attention until its full genome sequence was determined [77]. Since then, this virus has been detected in mosquitoes from multiple geographical regions, including Puerto Rico [78], Thailand [79], Mexico [80], Australia [81], and Brazil [82]. Studies of *Ae. aegypti* have shown that although it can be transmitted horizontally, vertical transmission appears to be the main route [83]. Amino acid sequence analysis between CFAV and MBFs revealed an average identity of 31.8% for NS3 and 44.3% for the NS5 protein which indicates substantial divergence from MBFs [79]. Superinfection studies with CFAV demonstrated that it can inhibit other human pathogenic orthoflaviviruses, such as dengue virus serotype 1 (DENV-1) and ZIKV, suggesting that it can be potentially used for reducing *Orthoflavivirus* transmission [84].

Kamiti River virus (KRV) was the second identified cISF, being first isolated from field-collected *Ae. macintoshi* mosquitoes from Kenya in 1999 [85]. KRV is closely related to CFAV according to the comparison of genome sequences [85,86], although, unlike CFAV, it lacks the ability to cause cell fusion [87]. The study of KRV transmission routes, conducted with *Ae. aegypti* mosquitoes in laboratory conditions, revealed that, similarly to CFAV, it is transmitted vertically [86].

Culex flavivirus is another cISF discovered at the early stages of research into ISFs. It is closely related to CFAV and KRV and is also vertically transmitted [88]. It was first isolated from *Cx. pipiens* mosquitoes from Japan [88] and later found in Guatemala [89], United States [90], Trinidad [90], Mexico [91], China [92], and Argentina [93]. Since then, many other ISF species have been discovered in different mosquito species from various geographical locations. This includes QBV isolated from *Cx. tritaeniorhynchus* and *Anopheles sinensis* mosquitoes collected in China and Vietnam, Calbertado virus detected from *Cx. tarsalis* mosquitoes in North America [94], Parramatta River virus (PaRV) isolated from *Ae. vigilax* mosquitoes [95], and Palm Creek virus (PCV) isolated from *Coquillettidia xanthogaster* mosquitoes [38] in Australia. Another cISF, NIEV, was isolated from the *Culex* mosquitoes collected in Côte d’Ivoire, West Africa, and its host range was evaluated in comparison with YFV, showing that it is restricted to replicate in insect hosts [54].

The transmission studies on cISFs demonstrated that natural circulation of cISF relies heavily on vertical transmission [83,86,88] by which an infected female directly conveys the virus to her offspring [96,97]. Transovarial (also known as transovarian) transmission is the most common type of vertical transmission of cISFs in which the virus is transmitted from a parent to its progeny via the ovaries. The infected female mosquito lays eggs that carry the virus, resulting in offspring that are born pre-infected upon hatching [98,99]. This transmission mechanism has been shown for CxFV [99], CFAV [83], Aedes flavivirus (AeFV) [100], and KRV [86]. Some ISFs can also be transmitted horizontally trough venereal (sexual) transmission. The assessment of venereal transmission of CxFV by mating of CxFV-infected male mosquitoes with uninfected females suggested that this transmission route plays a minor role for persistence of CxFV in mosquito populations [101]. In contrast, the transmission study of AeFV demonstrated that transovarial transmission is rare, while the venereal pathway is the main transmission route for AeFV [100]. Collectively, these studies indicate that transmission pathways of cISFs can be species-specific, although the majority of the species assessed to date rely on the vertical transovarial mechanism.

The host range of cISFs is restricted to insects, primarily mosquitoes and sand flies, and they cannot infect vertebrate hosts or replicate in vertebrate cell lines in vitro. The replication ability of CxFV has been assessed in different cell lines, showing that CxFV is unable to replicate in African green monkey kidney cells (Vero), chicken embryo fibroblast cells (DF-1) [102], and baby hamster kidney cells (BHK-21) [88]. Similarly, CxFV-related cISFs Spanish Ochlerotatus flavivirus and Spanish CxFV also showed no replication in Vero or BHK-21 cells [103]. CFAV also failed to replicate in Vero-E6 and BHK-21 cells [85]. Likewise, QBV was unable to replicate in the mammalian cell lines Vero, Rhesus monkey kidney cells (LLC-MK) strain 2, and BHK cell lines [104]. More recently, a comprehensive in vitro study based on PCV also demonstrated the restricted host range of the virus. PCV replication was assessed in Vero, BHK-21, porcine stable equine kidney (PS-EK), human adenocarcinoma (SW-13), and *Ae. albopictus* mosquito (C6/36) cell lines, and viral propagation was only observed in C6/36 cells with no evidence of infection in any vertebrate cell line [38]. Collectively, these findings demonstrate that all cISFs currently tested lack the ability to infect vertebrate cells, while their capacity to replicate in C6/36 has been consistently evident [95,105,106,107,108].

### 3.2. Lineage II or Dual-Host-Associated Insect-Specific Flaviviruses

In comparison to cISFs, dISFs are less extensively studied and characterised; however, the number of identified dISFs has increased in the recent two decades. In 2009, two strains of Nounané virus (NOUV) were isolated from *Uranotaenia mashonaensis* mosquitoes collected in Taï National Park, Côte d’Ivoire. These isolates showed high sequence identity at both nucleotide and amino acid levels, and phylogenetic investigations of the full polyprotein and the NS3 region confirmed their distinction from MBFs [109]. In the same year, Lammi virus (LAMV) was isolated in Finland. Closely related to NOUV, LAMV has shown through complete coding sequence analysis to be clearly distinct from human-pathogenic orthoflaviviruses [110]. Also in 2009, Chaoyang virus (CHAOV) was isolated from *Ae. vexans* mosquitoes in Liaoning Province, China [111], and later from *Ae. vexans nipponii* in South Korea [112]. The nucleotide sequence of CHAOV exhibited around 60% identity to human-pathogenic MBFs, positioning it close to MBFs in the phylogeny [111,113]. In the same year, Nanay virus (NANV) was isolated from *Cx. ocossa* mosquitoes in Peru, and although closely related to MBFs, NANV remains distinct from other *Orthoflavivirus* species [114]. Subsequent polyprotein amino acid sequence alignments revealed 53% identity and 70% identity between NANV and NOUV, typical dISFs [115]. Since then, more dISFs have been identified worldwide, including Ilomantsi virus isolated in Finland [116], Nhumirim virus (NHUV) isolated in Brazil [117], as well as Binjari virus (BinJV) [118] and Hidden Valley virus [118] in Australia.

To date, transmission studies have been conducted for only a limited number of dISFs. Nhumirim virus (NHUV) was intra-thoracically injected into female *Cx. pipiens* mosquitoes, and the progeny of the injected females tested NHUV-positive via RT-PCR and indirect fluorescent antibody assay (IFA). However, due to the restricted quantity of samples, vertical transmission could not be definitively established in this study [119]. The transovarial transmission of CHAOV was assessed in female *Ae. aegypti* mosquitoes that were intrathoracically injected with the virus. The viral RNA in offspring was quantified by real-time RT-PCR, demonstrating a high filial infection rate. The venereal transmission for CHAOV was assessed by mating infected female *Ae. aegypti* mosquitoes with healthy males, which revealed rare transmission [120]. In addition, LAMV was detected in the larvae, pupae, and adults of *Ae. cinereus* mosquitoes, demonstrating the possibility of transovarial transmission [121]. Collectively, these studies indicate that, similar to cISFs, circulation of dISFs is primarily supported by vertical transmission.

Although dISFs are phylogenetically related to MBFs, they exhibit the same restricted host range as cISFs, being unable to replicate in vertebrate cells [3]. For instance, NOUV infection was tested in multiple vertebrate cell lines, including Vero, BHK, human embryonic kidney (HEK293), human lung adenocarcinoma epithelial (A549), human epidermoid carcinoma Hep2, PS-EK, primary chicken embryo fibroblasts (CEF), and C6/36 cells. NOUV failed to replicate in all tested vertebrate cells but was successfully propagated in C6/36 cells [109]. In another study, in vitro culture of LAMV was attempted in primary CEF, human neuroblastoma SH-SY5Y, HEK293, human cervical carcinoma HeLa, mouse neuroblastoma Neuro-2A, BHK-21 hamster cells, porcine kidney PK-15, and Vero cells [110]. LAMV failed to establish replication in all of these mammalian cell lines as evidenced by the lack of cytopathic effect (CPE) or IFA-positive cells [110]. Viral culture of NANV in Vero-76, Vero-E6, BHK, LLC-MK, Madin–Darby canine kidney, A549, and human embryo rhabdomyosarcoma cells also failed, with all cultures negative by IFA and RT-PCR and showing no CPE [114]. These studies strongly support that, despite high sequence identity to MBFs, dISFs are unable to infect vertebrate cells across different species and remain fully restricted to replication in mosquito cells. High homology between dISF and MBF genome sequences together with strict host restriction enables the design of the chimeric viruses that express structural proteins of pathogenic MBFs in ISF backbones [23,54]. Such viruses can elicit neutralising antibodies against MBFs in mammals, while being uncapable of productive infection in vertebrate host. This has been recently used for successful design of the safe live attenuated vaccine [23] and has prompted further research interest into ISFs.

## 4. Mosquito Antiviral Strategies

Mosquitoes employ a multi-layered defence strategy against viral infections, encompassing physical and physiological barriers and innate immune responses [122,123]. Understanding how these antiviral mechanisms operate is essential for identifying the processes that determine viral competition, vector competence, and SIE. Physical barriers of mosquitoes include the exoskeleton, cuticle, trachea, midgut, haemocoel, fat body, and salivary glands, with the latter four also functioning as physiological barriers that initiate internal processes upon viral infection. However, the primary physiological barriers reside within the midgut and salivary glands, specifically the midgut infection barrier (MIB), midgut escape barrier (MEB), salivary gland infection barrier (SGIB), and salivary gland escape barrier (SGEB) [122,123]. Mosquitoes lack an adaptive immune system, and their physiological barriers for viral infection rely solely on the innate immune pathways [122]. The main and most potent immune pathway in mosquitoes is the RNA interference (RNAi) [124]. Additionally, infected mosquitoes activate transcription of innate immune genes encoding for antimicrobial peptides (AMPs) in the processes coordinated by three major signalling pathways, namely the Toll pathway, immune deficiency (IMD) pathway, and Janus kinase-signal transducer and activator of transcription (JAK-STAT) pathway. In this section, we will focus on major physiological barriers and immune signalling pathways that mediate antiviral response through immune signalling pathways that can be related to SIE.

### 4.1. RNA-Interference Pathway

**Figure 3:**
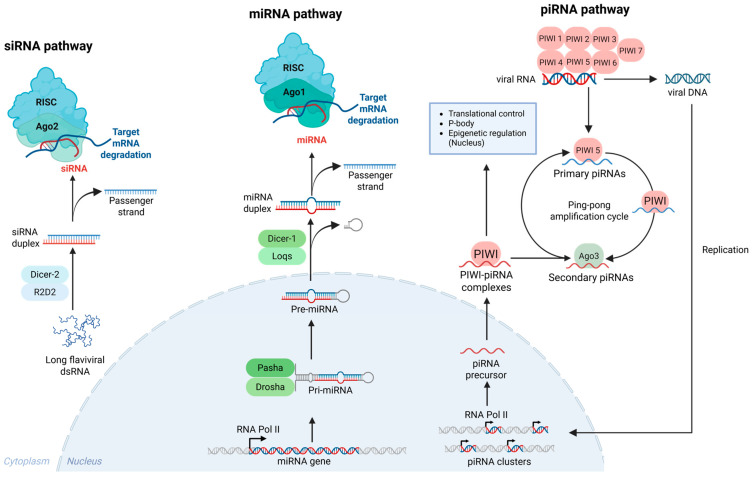
Overview of RNAi pathway during viral infection in mosquitoes. In the siRNA pathway, viral dsRNA intermediates are detected in the cytoplasm by the Dicer-2 and R2D2 complex, which cleaves them into ~21-nt siRNAs. These siRNAs are incorporated into RNA-induced silencing complex (RISC), where R2D2 assists in transferring the guide strand to Ago2 which targets and cleaves complementary viral mRNAs, thereby suppressing viral replication. In the miRNA pathway, small endogenous non-coding RNAs are transcribed by RNA polymerase II into pri-miRNAs in the nucleus. Subsequently, Drosha and Pasha process pri-miRNAs into pre-miRNAs which get exported to the cytoplasm. Thereafter, Dicer-1 and Loqs cleave pre-miRNAs into miRNA duplexes and are loaded onto RISC where Ago1 subsequently cleaves complementary host mRNAs or modulates their translation. In the piRNA pathway, viral RNA in the cytoplasm can undergo reverse transcription and integrate into the host genome within the nucleus, or it can associate with specific PIWI proteins (PIWI 1–7) and directly enters the ping-pong amplification cycle to bind to PIWI 5 for trimming and methylation to generate primary piRNAs. In this pathway, primary piRNAs bind to unidentified PIWI proteins and subsequently to Ago3, where trimming and methylation generate secondary piRNAs that associate with PIWI 5 to continue the ping-pong cycle. In the other pathway, a viral RNA strand can also undergo reverse transcription and replication before entering the host nucleus where it integrates into the host genome. Thereafter, it gets transcribed by host RNA polymerase II and translocated into the cytoplasm where it also participates in the ping-pong cycle. Created in BioRender. Goh, J. (2026). https://BioRender.com/0wd9v8r.

RNAi is a highly conserved sequence-specific gene-silencing mechanism operating at the post-transcription level that relies on small 18–30 nt RNAs. These RNAs are classified into three distinct groups based on their biogenesis, processing, and mechanism of action: small interfering RNAs (siRNAs), microRNAs (miRNAs), and P element-induced wimpy testis (PIWI)-interacting RNAs (piRNAs) (Figure 3) [124].

Among the small RNA groups, siRNAs are the most extensively studied and are responsible for a significant portion of the antiviral response [125]. Most mosquito-infecting viruses are either positive-sense (+) or negative-sense (−) ssRNA viruses, which generate double-stranded RNA (dsRNA) intermediates during replication [126]. The long viral dsRNA intermediates are recognised and bound by the RNA-binding domain of a complex consisting of RNase III endonuclease enzyme Dicer-2 and the R2D2 protein within the cytoplasm [127]. Subsequently, the dsRNA is cleaved into siRNAs, approximately 21 nucleotides in length, and loaded into the multiprotein RNA-induced silencing complex (RISC). RISC then unwinds the siRNA duplex, degrading the passenger strand while retaining the guide strand. R2D2 plays a crucial role in this process by facilitating the delivery of the siRNA guide strand from Dcr2 to Ago2. This ssRNA guide strand then directs RISC to complementary mRNA sequences in the viral genome, which are subsequently cleaved by the host endonuclease Ago2 [128]. This sequence of events constitutes the core of the siRNA-mediated antiviral defence in mosquitoes, enabling precise and efficient degradation of viral RNA to suppress infection.

MicroRNAs (miRNAs) are the small endogenous non-coding RNAs that, unlike siRNAs, are encoded by the host genome. They are transcribed into primary miRNAs (pri-miRNAs) containing stem-loop structures by host RNA polymerase II within the nucleus [129,130]. Subsequently, the ribonuclease III enzyme Drosha, in conjunction with the accessory protein Pasha, cleaves the pri-miRNA at the stem loop, generating the precursor miRNA (pre-miRNA) [131,132]. The pre-miRNA is then exported into the cytoplasm, where Dicer-1, along with the double-stranded RNA-binding protein Loquacious (Loqs), excises the terminal loop, forming a miRNA duplex that is subsequently loaded onto the RISC [133]. RISC then unwinds the miRNA duplex, degrading the passenger strand while retaining the guide strand. This ssRNA guide strand directs RISC to complementary mRNA sequences, which are subsequently cleaved by the host endonuclease Argonaute-1 (Ago1) [134,135]. Although miRNAs do not directly target viral genomes [136], they have been implicated in antiviral response in mosquitoes through regulation of antiviral genes and genes required for viral replication. For instance, *Ae. aegypti* miRNA miR-2940 was shown to be downregulated in response to WNV to restrict viral replication [137].

PIWI-interacting RNAs (piRNAs) are a class of small RNAs typically 24–31 nucleotides in length that associate with a subfamily of the Argonaute proteins called PIWI proteins. Unlike siRNAs or miRNAs, piRNAs are generated in a Dicer-independent manner from long single-stranded precursor transcripts [138]. Current understanding of piRNAs in mosquitoes remains limited. They are known to originate from diverse sources such as repetitive genomic sequences (piRNA clusters), virus-derived piRNAs (vpiRNAs), and viral mRNA [139]. piRNAs function in post-transcriptional gene silencing by targeting and degrading transposon transcripts within the cytoplasm, thereby preventing their translation [140].

During RNA virus infection in mosquitoes, viral RNA present in the cytoplasm can associate with specific PIWI proteins (PIWI 1–7) and enter the ping-pong amplification cycle for generation of piRNAs. During this process, the RNA binds to PIWI 5 for trimming and methylation, generating primary piRNAs [141,142]. These primary piRNAs then interact with unidentified PIWI proteins and subsequently with Ago3, where further trimming and methylation generate secondary piRNAs that, again, associate with PIWI 5 to sustain the ping-pong cycle [122,143]. In the alternative route, viral RNA may undergo reverse transcription and replication before integrating into the host genome within the nucleus [122,141]. The integrated sequences are then transcribed by host RNA polymerase II, and the resulting transcripts are exported to the cytoplasm, where they also participate in the ping-pong amplification process [122,141].

A hallmark of the primary piRNAs is the presence of a uridine at the first nucleotide position, referred to as the 1U bias [144]. Conversely, a defining feature of secondary piRNAs is their 10-nucleotide overlap with primary piRNAs at the 5′ end, coupled with the presence of an adenine at the 10th nucleotide position, referred to as the 10A bias [144]. Together, both 1U and 10A bias serves as molecular signatures of an active antiviral or transposon-silencing piRNA pathway [145,146]. Notably, Aag2 cells persistently infected with CFAV found a clear 10A and 1U bias [147], while it was not evident in PCV-infected *Ae. aegypti* mosquitoes [148]. A few studies of DENV infection in Aag2 cells and *Aedes* mosquitoes have found little evidence of a 10A or 1U bias [142,149,150]. Furthermore, DENV and ZIKV studies involving knockdown of *Ago3*, *Piwi5* or *Piwi6* did not significantly increase DENV-2 RNA levels or ZIKV titers and RNA levels, suggesting that the piRNA pathway may have limited or no antiviral effect in *Ae. aegypti* [142,151]. The *Ae. aegypti* genome encodes eight PIWI family proteins (PIWI 1–7 and Ago3), whereas *Ae. albopictus* possesses seven (PIWI 1–6 and Ago3) [152,153]. Consequently, it is hypothesised that different mosquito species utilise distinct PIWI pathway mechanisms to interact with diverse viral types [123,154].

### 4.2. JAK-STAT Pathway

**Figure 4:**
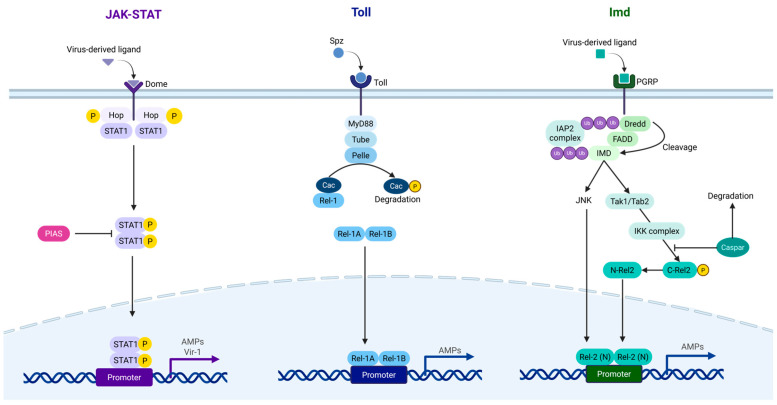
Overview of JAK/STAT, Toll, and IMD pathways during viral infection in mosquitoes. The JAK-STAT pathway is activated when a ligand binds to the Dome receptor, triggering its dimerisation and activation of the kinase Hopscotch (Hop). Phosphorylated Hop and Dome recruit and phosphorylate STAT proteins, which dimerise and translocate to the nucleus to regulate antiviral genes such as vir-1. PIAS acts as a negative regulator by inhibiting STAT phosphorylation and nuclear translocation. In the Toll pathway, virus-derived ligands are recognised by pathogen recognition receptors (PRRs), leading to the proteolytic cleavage of the cytokine Spätzle (Spz). The cleaved Spz binds to the Toll receptor, initiating a signalling cascade that recruits MyD88, Tube, and the kinase Pelle. This results in phosphorylation and degradation of the inhibitor Cactus, allowing the NF-κB transcription factors Rel1A and Rel1B to translocate into the nucleus and activate expression of effector genes, including antimicrobial peptides (AMPs). Similarly to the Toll pathway, the IMD pathway is triggered by the recognition of virus-derived ligands by PRRs which recruit adaptor proteins IMD, FADD, and Dredd. Activated Dredd, through ubiquitination by the IAP2 complex, cleaves and ubiquitinates IMD. This leads to the activation of the JNK signalling pathway and the recruitment of the Tak1/Tab2 and IKK complexes. The IKK complex phosphorylates and cleaves NF-κB transcription factor Rel2, which then translocates to the nucleus to induce expression of the effector genes such as AMPs. Degradation of the negative regulator Caspar, analogous to Cactus in the Toll pathway, further facilitates Rel2 activation. Created in BioRender. Zhao, Z. (2026). https://BioRender.com/9i1yct0.

The JAK-STAT pathway is the antiviral signalling cascade of insects that is similar to the interferon pathway of vertebrates. A schematic of the JAK-STAT pathway is shown in Figure 4. Activation begins by the binding of a ligand to the extracellular domain of the transmembrane receptor Dome, inducing a conformational change from a monomer to a dimer [155]. Currently, the specific ligands involved in JAK-STAT pathway activation in mosquitoes remain unidentified. However, in *Drosophila*, three Unpaired proteins (Upd1, Upd2, Upd3) fulfil this role [156]. Dome activation leads to the self-phosphorylation of the receptor-associated kinase Hopscotch (Hop), which subsequently phosphorylates the cytoplasmic region of the Dome receptor. This creates a docking site for the SH2 domain of STAT proteins [155,157]. STAT proteins, existing as inactive monomers in the cytoplasm, bind to the phosphorylated cytoplasmic region of the Dome receptor, resulting in phosphorylation and dimerisation [155]. Phosphorylated STAT dimers are then translocated to the nucleus, where they bind to palindromic sequences within the promoters of target genes, such as virus-induced RNA 1 (vir-1), which plays a crucial role in antiviral responses [155,158]. Furthermore, the binding of the secretory Vago protein to an unidentified transmembrane receptor has been shown to activate the JAK-STAT pathway following WNV infection in *Cx. quinquefasciatus*, suggesting that different viruses may activate this pathway through different cytokine receptors [159].

### 4.3. Toll Pathway

Activation of the Toll pathway is initiated by the binding of virus-derived ligands to pathogen recognition receptors (PRRs), resulting in the cleavage of the cytokine Spätzle (Spz) [160]. The cleaved Spz acts as a ligand, binding to the Toll transmembrane receptor and triggering a signalling cascade [160]. This leads to the recruitment of adaptor proteins MyD88, Tube, and the kinase Pelle to the receptor, resulting in the phosphorylation and proteasomal degradation of the negative regulator Cactus [161,162]. Cactus normally sequesters the nuclear factor kappa B (NF-κB)-like transcription factor Rel1 in the cytoplasm [162]. Degradation of Cactus allows the translocation of Rel1A and its co-activator Rel1B from the cytoplasm to the nucleus, where they bind to κB-motifs on the promoters of effector genes, such as antimicrobial peptides (AMPs) [163]. In *Ae. aegypti*, nine Toll receptors have been identified, although their specific functions remain largely unknown. Recent studies in *Ae. aegypti* and *Cx. quinquefasciatus* have suggested that Toll receptor 6 may initiate Toll pathway activation by directly binding to double-stranded RNA (dsRNA) within the endosomes [164,165].

### 4.4. IMD Pathway

Similarly to the Toll pathway, the IMD pathway is initiated by the binding of virus-derived molecules to PRRs. Activated PRRs recruit adaptor proteins IMD, FADD, and Dredd [122,166,167]. Dredd undergoes ubiquitination and activation by the IAP2 complex. Ubiquitinated Dredd cleaves IMD and is subsequently ubiquitinated by the IAP2 complex [168]. Ubiquitinated IMD activates the JNK signalling pathway which induces production of AMPs and has been shown to reduce DENV-2, ZIKV, and CHIKV infection in *Ae. aegypti* [169]. Additionally, ubiquitinated IMD also recruits intermediate signalling complex Tak1/Tab2, which subsequently recruits and activates the IKK phosphorylation complex [170]. This complex then phosphorylates NF-κB transcription factor Rel2 where it subsequently gets cleaved. Additionally, the recruitment of the adaptor proteins IMD, FADD, and Dredd results in the degradation of the negative regulator Caspar, mirroring the role of Cactus in the Toll pathway, and also contributes to phosphorylation and cleavage of Rel2 [124,171,172]. Subsequently, Rel2 is translocated from the cytoplasm to the nucleus for the transcription of IMD-related genes such as AMPs [167].

Together, the innate immune pathways in mosquitoes constitute a dynamic hierarchical antiviral defence system that plays a crucial role in regulating arboviral infections and may contribute to shaping the SIE outcomes. By establishing a hostile intracellular and extracellular environment during primary infection, these antiviral mechanisms may not only suppress initial viral replication but also create physiological conditions that impede superinfection by secondary viruses. Therefore, the interplay between immune signalling and physical compartmentalisation is consider as one of the potential foundations for SIE in mosquitoes [173].

## 5. Superinfection Exclusion Capacity of ISFs

Superinfection exclusion is a phenomenon where a pre-existing (primary) viral infection within a host inhibits or significantly reduces secondary viral infection [25,26]. To date, the capacity for the homologous SIE has been tested for AEFV, BinJV, CFAV, CxFV, LAMV, NHUV, PCV, PaRV, and QBV in in vitro and in vivo systems (Table 1 and Table S1). 

**Table 1:** Homologous superinfection exclusion studies in orthoflaviviruses.

Primary ISF (Country) Dose	Secondary MBF (Country) Dose	Model	Key Findings	Reference
AEFV (China) 6 × 10^6^ genome copies/µL	ZIKV_MR766_ (East Africa) Blood fed 1 × 10^5^ FFU/mL	*Ae. albopictus* * (In vivo)	ZIKV RNA significantly lower at 7 dpi with no significant difference at 3, 7, and 12 dpi.	[105]
	DENV-2_NGC_ (New Guinea) Blood fed 1 × 10^8^ FFU/mL		DENV-2 RNA significantly lower at 7 dpi with no significant difference at 3 and 12 dpi.	
BinJV_BFTA20_ (Australia) MOI = 1	WNV_578/10_ (Hungary) MOI = 1	C6/36 (In vitro)	WNV titre significantly lower at 1–4 dpi.	[174]
BinJV_BFTA20_ (Australia) persistently infected			WNV titre significantly lower at 3–4 dpi.	
BinJV_BFTA20_ (Australia) MOI = 1	ZIKV_NL00013_ (Suriname) MOI = 1		ZIKV titre significantly lower at 2–4 dpi.	
BinJV_BFTA20_ (Australia) persistently infected				
BinJV_BFTA20_ (Australia) MOI = 1	WNV_578/10_ (Hungary) MOI = 1	Aag2 (In vitro)	WNV titre significantly lower at 3 dpi but similar at 4 dpi.	
BinJV_BFTA20_ (Australia) persistently infected			WNV titre significantly lower at 2–3 dpi but similar at 4 dpi.	
BinJV_BFTA20_ (Australia) MOI = 1	ZIKV_NL00013_ (Suriname) MOI = 1		ZIKV titre significantly lower at 2–4 dpi.	
BinJV_BFTA20_ (Australia) persistently infected				
BinJV_BFTA20_ (Australia) MOI = 1		Aag2 Ago2 deficient (In vitro)		
BinJV_BFTA20_ (Australia) MOI = 1	ZIKV MOI = 1 *	C6/36 (In vitro)	ZIKV titre significantly lower at 1–5 dpi.	[175]
CFAV * #	DENV-2 MOI = 1 *	Aa20 (In vitro)	DENV-2 titre significantly higher at 3 dpi.	[176]
			DENV-2 RNA significantly higher at 3 dpi.	
CFAV (Thailand) MOI = 0.23	DENV-1 (Thailand) MOI = 0.1	C6/36 (In vitro)	Increased reduction in DENV-1 titre as CFAV and DENV-1 time interval increased.	[84]
CFAV (Thailand) MOI = 0.11	ZIKV (French Polynesia) MOI = 0.1		ZIKV titre significantly lower at 4–7 dpi.	
CFAV (Thailand) 1.14 × 10^7^ TCID50/mL	DENV-1 (Thailand) Blood fed 5 × 10^6^ FFU/mL	*Ae. aegypti*, Thailand (In vivo)	DENV-1 dissemination rate marginally significantly lower at 2–13 dpi. DENV-1 titre significantly lower at 13 dpi.	
	ZIKV (French Polynesia) Blood fed 7.5 × 10^6^ FFU/mL		ZIKV dissemination rate no detectable difference at 13 dpi. ZIKV titre significantly lower at 13 dpi.	
CFAV 5.39 × 10^6^ TCID50/mL *	DENV-1 (Thailand) Blood fed 5 × 10^6^ FFU/mL		DENV-1 dissemination rate no detectable difference at 13 dpi. DENV-1 titre significantly lower at 13 dpi.	
CxFV (USA) 0.1 RNA copy/well	WNV (USA) 0.1 PFU/well	C6/36 (In vitro)	WNV titre significantly lower at 60, 108–156 hpi but equivalent at 168 hpi.	[101]
	WNV (USA) 0.01 PFU/well		WNV titre significantly lower at 84–156 hpi but equivalent at 168 hpi.	
CxFV (USA) naturally infected	WNV (USA) Blood fed 1 × 10^7^ PFU/mL	*Cx. pipiens*, USA (In vivo)	WNV transmission and infection rate no detectable difference at 7 and 14 dpi. WNV dissemination rate significantly lower at 7 dpi no detectable difference at 14 dpi.	
			WNV RNA no detectable in bodies and saliva at 7 and 14 dpi. WNV RNA significantly lower in legs, heads and wings at 7 dpi and no detectable difference at 14 dpi.	
CxFV_NIID21-2_ (Japan) persistently infected	JEV_Mie/41/2002_ (Japan) 0.1 PFU/cell	NIID-CTR (In vitro)	JEV titre significantly higher at 6 dpi.	[177]
	DENV-2_NIID02-20_ (Thailand) 0.1 PFU/cell		DENV-2 titre significantly higher at 5–7 dpi.	
CxFV_IzabalGU-06-2692_ (Guatemala) MOI = 0.1	WNV_GU-06-2256_ (Guatemala) MOI = 0.1	C6/36 (In vitro)	WNV titre no significant difference at 1–14 dpi.	[178]
CxFV_IzabalGU-06-2692_ (Guatemala) 1 × 10^3.3^ PFU	WNV_GU-06-2256_ (Guatemala) Blood fed 1 × 10^6.3^ PFU/mL	*Cx. quinquefasciatus*, USA (In vivo)	WNV titre significantly higher at 4 dpi with no significant difference at 1, 2, 8, and 10 dpi.	
CxFV_IzabalGU-06-2692_ (Guatemala) 1 × 10^2.8^ to 1 × 10^3.3^ PFU	WNV_GU-06-2256_ (Guatemala) Blood fed 1 × 10^8.9^ PFU/mL	*Cx. quinquefasciatus*, USA and Honduras (In vivo)	WNV titre no significant difference at 14 dpi. WNV transmission, infection, and dissemination rate, no significant difference at 14 dpi.	
	WNV_GU-06-2256_ (Guatemala) Blood fed 1 × 10^7.4^ to 1 × 10^7.5^ PFU/mL 1 × 10^5.4^ to 1 × 10^5.6^ PFU/mL for USA and Honduras mosquito respectively			
LAMV_2009/FI/Original_ (Finland) 1.75 × 10^5^ RNA copies/well	WNV-1 (Africa) MOI = 0.1	U4.4 (In vitro)	WNV titre significantly lower at 24–96 hpi.	[179]
NHUV (Brazil) MOI = 5	WNV_NY99_ (USA) MOI = 0.1	C6/36 (In vitro)	WNV, JEV, and SLEV titre significantly lower at 2–7 dpi.	[180]
	JEV MOI = 0.1 *			
	SLEV MOI = 0.1 *			
NHUV (Brazil) MOI = 1	WNV_NY99_ (USA) MOI = 0.1	C6/36 (In vitro)	WNV titre significantly lower at 1–7 dpi.	[119]
		C7-10 (In vitro)		
NHUV MOI = 5 *	ZIKV MOI = 0.1 *	C6/36 (In vitro)	ZIKV titre significantly lower at 2–7 dpi.	[181]
			ZIKV RNA significantly lower at 48–72 hpi.	
	DENV-2 MOI = 0.1 *		DENV-2 titre significantly lower at 2–7 dpi.	
NHUV 1 × 10^4^ PFU *	ZIKV Blood fed 1 × 10^2^ PFU *	*Ae. aegypti*, Mexico (In vivo)	ZIKV dissemination and infection rate significantly lower at 14 dpi. ZIKV titre no significant difference at 14 dpi.	
PaRV (Australia) MOI = 5	DENV-3 (Australia) MOI = 0.1	C6/36 (In vitro)	DENV-3 titre significantly lower at 48–96 hpi.	[182]
	WNV_KUNVMRM16_ (Australia) MOI = 0.1		WNV titre significantly lower at 24–96 hpi.	
PCV (Australia) MOI ≥ 1	WNV_KUNVMRM16_ (Australia) MOI = 0.1	C6/36 (In vitro)	WNV titre significantly lower at 24–48 hpi.	[38]
	MVEV MOI = 0.1 *		MVEV titre significantly lower at 24–48 hpi.	
PCV (Australia) 1 × 10^4^ TCID50/mL	WNV_KUN2009_ (Australia) Blood fed 1 × 10^7^ TCID50/mL	*Cx. annulirostris*, Boondall Wetlands, Hemmant and Tingalpa in Australia (In vivo)	WNV transmission and infection rate significantly lower with no significant difference in dissemination rate at 10–12 dpi. WNV titre no significant difference at 10–12 dpi.	[183]
	WNV_KUN2009_ (Australia) 1 × 10^5.7^ TCID50/mL	*Cx. annulirostris*, Boondall Wetlands in Australia (In vivo)	WNV transmission and infection rate no significant difference at 10–12 dpi. WNV titre significantly lower at 10–12 dpi.	
	WNV_KUN2009_ (Australia) 1 × 10^5^ TCID50/mL		WNV transmission and infection rate no significant difference at 10–12 dpi. WNV titre significantly higher at 10–12 dpi.	
PCV (Australia) 1 × 10^6^ TCID50/mL	ZIKV_MR766_ (East Africa) Blood fed 1 × 10^6^ PFU/mL	*Ae. aegypti*, Thailand (In vivo)	ZIKV transmission, infection and dissemination rate no significant difference at 14 dpi. ZIKV titre no significant difference at 10–12 dpi.	[184]
QBV_490 Cx.ge 9/2/21 T1_ (Singapore) MOI = 1	DENV-2_GII_ MOI = 0.1 *	C6/36 (In vitro)	DENV-2 titre significantly lower at 1–6 dpi.	[185]
	WNV_KUNVMRM16_ (Australia) MOI = 0.1		WNV titre significantly lower at 1–6 dpi.	

* refers to unknown country; # refers to unknown dose. Abbreviations: Viruses: AEFV–Aedes flavivirus, BinJV–Binjari virus, CFAV–Cell fusing agent virus, CxFV–Culex flavivirus, DENV–Dengue virus, JEV–Japanese encephalitis virus, LAMV–Lammi virus, NHUV–Nhumirim virus, PaRV–Parramatta River virus, PCV–Palm Creek virus, SLEV–Saint Louis encephalitis virus, WNV–West Nile virus, WNV_KUN_–West Nile virus Kunjin strain, ZIKV–Zika virus; Experimental terms: dpi–days post-infection, hpi–hours post-infection, FFU–Focus-forming units, MOI–Multiplicity of infection, PFU–Plaque-forming units, TCID50–50% tissue culture infectious dose.

### 5.1. Superinfection Exclusion In Vitro

The ability of various ISFs to cause SIE has been extensively studied in mosquito cell lines. Importantly, these studies revealed that the use of different strains of primary ISFs and secondary MBFs can lead to varying outcomes in homologous SIE. For example, Baidaliuk et al. (2019) reported a reduction in DENV-1 replication in CFAV-infected C6/36 cells, whereas Zhang et al. (2017) observed a 4-fold increase in replication of DENV-2 in CFAV-infected Aa20 (*Ae. aegypti*) cells [84,176]. Notably, Baidaliuk et al. (2019) used a CFAV strain phylogenetically clustered with those naturally circulating in Thai mosquito populations, while Zhang et al. (2017) employed a genetically divergent Aag2 cell-derived CFAV strain [79,84,176]. The CFAV strain from Aag2 cells carries several premature stop codons that disrupt the codon-overlapping gene *fifo* which indicates that it is genetically different to the naturally circulating strain [53]. Importantly, an in vivo study by Hall-Mendelin et al. (2016) demonstrated that infection with the same PCV strain but different WNV strains (WNV_KUN2009_ and WNV_KUNMRM16_) led to opposite effects: WNV_KUN2009_ replication was significantly reduced, while WNV_KUNMRM16_ replication was significantly enhanced in mosquito bodies [183]. These findings collectively indicate that the genetic variation within both primary ISFs and secondary MBFs can substantially affect the outcomes of superinfection.

Additionally, analysis of the published in vitro studies suggests that the outcomes of *Orthoflavivirus* superinfection are influenced not only by intrinsic viral genetic factors but also by external variables and experimental designs. The inoculating dose between the primary ISF and secondary MBF tend to constitute another factor that can affect the outcome of superinfection. Most homologous SIE studies used either the same multiplicity of infection (MOI) for the primary infecting ISF and secondary infecting MBF or higher MOI for the primary infecting ISF (Table 1). Bolling et al. (2012) infected C6/36 cells with CxFV at a dose of 0.1 RNA copy/well and challenged with WNV 48 h later at two different doses of 0.01 pfu/well and 0.1 pfu/well [101]. In CxFV-infected cells challenged with WNV at 0.1 pfu/well, WNV titres were significantly lower at 60 h post-infection (hpi) and between 108 and 156 hpi compared to WNV-only control, while no significant difference was observed between 72 and 96 hpi. In contrast, when the cells were challenged with WNV at 0.01 pfu/well, the significant difference was only evident between 84 and 156 hpi. It is possible that a higher MOI of the primary infecting ISF could lead to a SIE effect at earlier time points but a delayed effect at later time points. A co-infection study by Romo et al. (2018) in C6/36 cells showed a 10-fold reduction in ZIKV replication when cells were co-infected with NHUV at an MOI of 1.0 and ZIKV at an MOI of 0.1, compared to co-infection with both viruses at an MOI of 0.1 [181]. The study also found an approximate 30-fold reduction in ZIKV replication when comparing ZIKV at MOIs of 0.1 and 1.0, both co-infected with NHUV at an MOI of 1.0. However, it is important to note that these results were from a co-infection study with inoculation of both viruses performed simultaneously [181]. This may not accurately represent natural superinfection patterns in which exposure to different viruses occurs sequentially.

The time interval between the primary ISF infection and the secondary MBF infection can also affect the outcomes of *Orthoflavivirus* superinfection. Baidaliuk et al. (2019) showed an increased reduction in DENV-1 titres in vitro in C6/36 cells as the time interval between the initial CFAV inoculation and the secondary DENV-1 infection increased [84]. However, when performed in vivo in *Ae. aegypti* mosquitoes, no effect of the time interval between primary and secondary infection on DENV-1 replication was evident [84]. Another superinfection study with different DENV serotypes in C6/36 cells showed that the longer the time interval between the primary and secondary infecting DENV strains, the greater the suppression on the replication of the superinfecting virus, with the caveat that this was performed using DENV instead of ISF as the primary infecting virus [186].

Although in vitro experiments have provided substantial insights into the ability of ISFs to induce SIE, it is important to recognise the limitations of these studies, which were conducted primarily in *Aedes* cell lines such as C6/36, Aa2, and Aag2. C6/36 cells lack functional siRNA-mediated RNAi due to the absence of Dicer-2 activity [187,188]. Although this makes them permissive for a wide range of viruses and a valuable tool for virology research, C6/36 cells may not accurately recapitulate the entire spectrum of the immune processes involved during *Orthoflavivirus* superinfection in RNAi-competent wild mosquitoes. Furthermore, a recent study demonstrated that acute ISF infection in immune-deficient cells such as C6/36 and Ago2-deficient Aag2 cells induced significant CPE, resulting in reduced cell viability and subsequently cell death, which likely influenced the viral replication dynamics of superinfecting MBFs [174]. Moreover, it has been well established that different cell lines harbour different adventitious viruses. For example, Aag2 cells are known to be persistently infected with CFAV and Phasi Charoen-like virus (PCLV), while C6/36 cells are sometimes infected with densoviruses [189,190,191]. Presence of the commensal viruses may affect the interactions between ISFs and MBFs in the superinfection studies, which should also be taken into account when extrapolating the results of the in vitro SIE experiments [34,188,192].

Additionally, most of the in vitro SIE studies utilised acute primary ISF infection (Table 1), which does not accurately reflect natural infection dynamics in mosquitoes. Mosquitoes are frequently persistently infected with ISFs from birth via vertical transmission routes, such as transovarial or transovum transmission [24]. So far, only a few studies examined *Orthoflavivirus* SIE in the context of persistent ISF infection [101,174]. In the in vitro study by Willemsen et al. (2025), C6/36 and Aag2 cells acutely infected with BinJV three days prior to secondary MBF infection showed stronger inhibition of WNV and ZIKV replication compared to cells persistently infected with BinJV [174]. Another study, which used NIID-CTR cells (*Cx. tritaeniorhynchus*) persistently infected with CxFV demonstrated a significant increase in JEV and DENV-2 replication following superinfection [177].

This finding indicates that persistent ISF infection can, in some cases, enhance secondary MBF replication, which further highlights the importance of understanding the mechanisms of SIE and viral interactions in mosquito vectors prior to implementation of ISF-based biocontrol strategies in the field.

Collectively, the results from the in vitro studies clearly demonstrate the capacity of multiple ISFs to induce SIE against MBFs. However, interpretation of homologous in vitro SIE experiments may be confounded by the characteristics of the laboratory cell lines and the experimental designs. This highlights the importance of validating the results of such studies in vivo and calls for employing or developing persistently infected cell lines or mosquito models that would provide a more biologically relevant model for *Orthoflavivirus* SIE studies.

### 5.2. Superinfection Exclusion In Vivo

To date, in vivo SIE experiments have been conducted for AEFV, CFAV, CxFV, NHUV, and PCV [84,101,105,178,181,183,184] (Table 1). Most vector competence studies indicate a competitive interaction between a primary ISF and a secondary MBF during early infection. However, no discernible effect of ISF on infection, dissemination, or transmission rates of MBF was observed at later time points after initial ISF inoculation [84,101,178,183,184].

When *Cx. quinquefasciatus* mosquitoes were intrathoracically injected with CxFV and superinfected with WNV via exposure to the infectious blood meal, no significant difference in WNV replication, dissemination, or transmission was observed between superinfected and WNV-only groups at 14 days post-infection (dpi) [178]. In another study, prior infection with PCV in *Ae. aegypti* followed by ZIKV superinfection resulted in no significant difference in ZIKV replication in midgut, body, and saliva, nor in infection, dissemination, or transmission rates at 14 dpi. However, a significant reduction in ZIKV replication was observed in mosquito heads at 14 dpi [184]. Likewise, prior infection with CxFV in *Cx. pipiens* followed by WNV superinfection resulted in no significant difference in WNV replication in bodies and saliva, nor in infection or transmission rates at 7 and 14 dpi. However, significantly lower WNV replication and dissemination were observed at 7 dpi, but not at 14 dpi, as assessed by WNV-positive legs, heads, and wings [101]. However, the authors have noted that the *Cx. pipiens* used in this study were from different geographical origins which may have affected vector competence results for WNV. This observation is further supported by the study that compared SIE in the laboratory-colonised and field-collected *Cx. annulirostris* [183]. When infected with PCV and subsequently blood fed with WNV_KUN2009_, significantly lower infection and transmission rates were observed at 10 to 12 dpi in the wild collected mosquitoes. However, when the laboratory-colonised *Cx. annulirostris* were infected with PCV and subsequently intrathoracically injected with either WNV_KUN2009_ or WNV_KUNMRM16_, no significant difference was evident in infection or transmission rate. In the study, the laboratory-colonised *Cx. annulirostris* has been in colony for over 50 generations as noted by the authors [183]. Laboratory colonisation can impact the genetic diversity and fitness of mosquito populations, potentially altering their vector competence compared to freshly collected field specimens [193]. Additionally, it is generally assumed that field-collected mosquitoes or stocks from other laboratory colonies (especially eggs) are virus-free, while wild mosquitoes in different geographic regions can be co-infected with various commensal viruses that may interfere with those used in SIE vector competence studies [194]. On the other hand, wild mosquitoes of the same species of different geographical origins have genetic divergence acquired over the course of co-evolution with different ISFs within each region [195]. For instance, mosquitoes of the same species but from different regions harbour different endogenous flaviviral elements (EFEVs) originated from persistent *Orthoflavivirus* infections [196]. In *Ae. albopictus*, the Vietnam strain contains *Ae. albopictus* flaviviral element (ALFE) 4 and ALFE7 with differences in ALFE1 as compared to the Gabon strain [196], while in *Ae. aegypti*, the full *Ae. aegypti* flaviviral element (AEFE) 1 was found in the French Guiana strain while only partial AEFE1 was found in the Cameroon and Thailand strains [196].

These EFEVs are likely involved in the piRNA pathway of the mosquito immune response during viral infection and therefore may affect vector competence in *Orthoflavivirus* superinfection [195,196,197].

Collectively, in vitro and in vivo studies demonstrate that homologous SIE of ISFs is influenced by various factors, including ISF and MBF strains, inoculation dose and timing, mosquito geographical origins, and presence of adventitious viruses. Further research utilising standardised in vivo models and persistently infected immunocompetent cell lines free of the commensal viruses is required for a more comprehensive understanding of this process.

## 6. Molecular Mechanisms of the ISF SIE

Homologous SIE is thought to be governed by an interplay of host and viral factors. Several studies hypothesised that RNAi could be one of the host factors responsible for the homologous SIE between ISFs and MBFs. It was suggested that sequence similarity between the primary infecting and secondary infecting virus can result in targeting of the MBFs by siRNA produced from the ISF genome [198,199]. A study by Willemsen et al. (2025) using Aag2 cells showed that the BinJV-ZIKV chimaera elicited potent SIE against ZIKV but not against WNV [174]. This selective effect was likely due to the generation of ZIKV-specific siRNAs, driven by the presence of the ZIKV prME sequence in the chimeric BinJV-ZIKV genome [174]. Another study performed by Koh et al. (2021) reported that prior infection of *Ae. aegypti* with PCV did not significantly affect ZIKV replication or vector competence, which was linked to the lack of the perfect 21 mer sequence identity between the PCV and ZIKV genomes [184]. In contrary, the in vivo study by Hall-Mendelin et al. (2016) demonstrated that although PCV shares slightly greater genomic identity with WNV_KUNMRM16_ than with WNV_KUN2009_, it enhanced WNV_KUNMRM16_ replication while suppressing WNV_KUN2009_ replication in mosquito bodies [183]. Furthermore, *Orthoflavivirus* SIE has been reported in C6/36 cells that lack functional RNAi response. For instance, the study by Goenaga et al. (2015) demonstrated significant reduction in WNV replication in C6/36 cells pre-infected with NHUV and superinfected with WNV [119,195]. Collectively, these studies suggest that SIE is unlikely to be driven by cross-targeting RNAi resulting from the sequence homology between the interacting viruses. On the other hand, ISFs may induce the JAK/STAT pathway, which can restrict replication of MBFs such as WNV [159], although this mechanism has not been directly demonstrated and further studies are required to elucidate the contribution of mosquito innate immune pathways to ISF SIE.

Another proposed mechanism of ISF SIE is the downregulation or internalisation of host cell surface receptors by the primary infecting virus, which reduces the availability of entry factors and prevents secondary viruses from gaining access to the same cell. Lee et al. (2005) demonstrated that, in pestiviruses, the structural glycoprotein E2 of primary infecting noncytopathic bovine viral diarrhoea virus (BVDV) is essential for the homologous SIE of secondary infecting cytopathic BVDV and that it acts by blocking viral entry [200]. Similarly, human immunodeficiency virus type 1 and influenza downregulate or internalise coreceptors, preventing attachment and entry of secondary infecting viruses [201,202]. However, whether ISFs cause similar depletion of the entry receptors used by MBFs is yet to be elucidated.

Competition for cellular resources can also constitute a mechanism of SIE as the infected cells are likely to be depleted of the metabolites required for viral replication or monopolised by the primary infecting virus, which would limit their permissiveness for subsequent infection. This mechanism is supported by the in vivo studies on WNV in *Culex* mosquitoes. In mosquitoes infected with PCV via vertical transmission, PCV was present in the midgut cells, where it replicated more efficiently than WNV [183]. In this study, PCV elicited potent SIE against WNV. It is likely that at the time of secondary infection the cellular resources in the midgut would have been depleted by PCV before subsequent WNV infection, resulting in homologous SIE. On the contrary, *Cx. quinquefasciatus* mosquitoes intrathoracically injected with CxFV and subsequently blood fed with WNV showed no significant differences in WNV replication compared to controls that received WNV alone [178]. It can be speculated that SIE was not observed because intrathoracic injection of CxFV bypasses the midgut barrier and prevents colonisation of the midgut by the virus. Therefore, WNV delivered via the oral route faces no competition in the midgut cells, which enables productive infection and dissemination of the virus [203].

Furthermore, the study with WNV replicons in baby hamster kidney cells (BHK-21) subsequently superinfected with WNV provided further evidence that intracellular host resources such as cellular proteins and lipid membrane components essential for RNA replication complex formation can become saturated during primary infection [204]. Notably, WNV replicons excluded replication of other orthoflaviviruses such as DENV-2, YFV, and SLEV but not non-orthoflaviviruses, suggesting competition for similar intracellular resources [204]. Similarly, another study found that the outer membrane region of JEV NS2B inhibits ZIKV replication [205]. NS2B is a key component of the *Orthoflavivirus* replication complex, playing an essential role in RNA replication and virion formation [206]. The outer membrane region of NS2B is oriented toward the cytoplasmic viral replication complex, suggesting that it competes with ZIKV for intracellular resources, thereby interfering and inhibiting ZIKV replication [207]. Additionally, studies with hepatitis C virus and BVDV have demonstrated that SIE can occur at the level of RNA replication and translation [200,208]. Moreover, some viruses remodel host membranes or organelles to form specialised replication niches, which can prevent secondary viruses from accessing the same intracellular compartments. For example, mutations in the WNV NS4A protein, which is crucial for endoplasmic reticulum membrane remodelling and replication complex formation, have been shown to enable the virus to bypass SIE in BHK-21 cells [209].

Overall, the studies conducted to date on the mechanisms of SIE in ISFs suggest that it is likely a multifactorial phenomenon. While sequence-specific cross-targeting of the secondary virus by small interfering RNAs does occur in some engineered systems, the cumulative evidence shows that it is neither necessary nor sufficient for SIE, as exclusion occurs even in RNAi-deficient cells and when sequence homology is lacking. Upregulation of innate antiviral responses such as the JAK/STAT pathway by the primary infecting ISF may potentially contribute to SIE but have not been directly demonstrated. The mechanism involving depletion of host factors and competition for replication resources currently has the best evidential support, with several studies indicating that by monopolising key cellular substrates and compartments required for *Orthoflavivirus* replication, a primary ISF can block secondary infection and replication of mosquito-borne viruses. Nevertheless, comprehensive in vivo studies are required to elucidate the relative importance of all proposed mechanisms.

## 7. Use of ISFs in SIE-Based Biocontrol Strategies

Current mosquito vector control for MBFs includes environmental management, such as eliminating breeding sites and modifying environments to reduce mosquito suitability. Chemical insecticides have also been extensively employed for the past century to suppress mosquito populations; however, their widespread use has driven the global emergence of insecticide-resistant mosquito strains and behavioural adaptations that reduce intervention efficacy [210,211]. Additionally, insecticide application poses significant risks to both environmental and human health [212]. The use of bed nettings is also ineffective against key diurnal flavivirus vectors like *Ae. aegypti* and *Ae. albopictus* [213,214]. Although these established methods have contributed to reducing disease transmission, their scalability, sustainability, and long-term effectiveness have been increasingly questioned over the past century. This led to the development and subsequent deployment of insect sterilisation techniques and Wolbachia-based strategies for mosquito vector control in numerous countries over the recent decades. The use of Wolbachia has been exceptionally successful in limiting DENV transmission, with multiple field studies conducted across various countries successfully demonstrating reduced viral transmission [215,216,217,218,219,220]. However, this approach remains labour-intensive and resource-demanding due to the need for precise sex-sorting technologies and logistical challenges associated with large-scale releases. Moreover, Wolbachia density and transmission efficiency are negatively affected by elevated temperatures, posing additional challenges under ongoing climate change [221,222]. Therefore, novel biocontrol strategies involving the genetic modification of mosquitoes have been developed to suppress or modify mosquito populations, while overcoming the limitations of the Wolbachia deployment. Non-gene drive genetic modification strategies such as self-limiting Release of Insects carrying a Dominant Lethal gene (RIDL) have been engineered in the transgenic Ae. aegypti OX513A strain for field studies in multiple countries with promising results [223,224,225,226,227,228,229]. Despite the advancements in RIDL, the monetary and fitness costs associated with mass-rearing mosquitoes using tetracycline, along with the fitness costs of using irradiation, prompts the use of alternative biocontrol strategies [230].

Due to their ability to elicit superinfection exclusion and minimal associated safety concerns, ISFs are currently considered as a promising new platform for the biocontrol of pathogenic mosquito-borne flaviviruses. Multiple studies have demonstrated that ISFs naturally infect mosquitoes and are unable to replicate in vertebrate hosts, which addresses a major safety consideration associated with the release of modified organisms [76,87,88,102,105,110,231]. Additionally, since ISFs are naturally present in wild mosquito populations, the regulatory hurdles are potentially lower than with genetically modified approaches [232]. Many ISFs establish persistent, low-fitness-cost infections in mosquitoes, and their ability to be vertically transmitted enables stable dissemination across mosquito generations [24,33,233,234]. Some ISFs such as CFAV have also been shown to be transmitted horizontally via venereal transmission, creating a self-sustaining system within mosquito populations [83]. This reduces the need for frequent and resource-intensive mosquito releases. Additionally, the use of naturally occurring ISFs may help avoid unintended disruptions to ecosystems that have evolved over long periods [235].

## 8. Conclusions

Despite extensive research and implementation of various current and novel vector control strategies, MBFs continue to cause outbreaks globally, highlighting the need for innovative control methods. Consequently, ISFs, with their ability to induce SIE while being incapable of replicating in vertebrate hosts, are now being considered for potential use as a biocontrol strategy against MBFs. Currently, most studies on SIE by ISFs remain at the proof-of-concept stage, with the majority being conducted in laboratory settings. These studies have revealed several important insights into the biology of SIE. They demonstrated that SIE is influenced by multiple factors such as virus genetics, superinfection timelines, and environmental variables. However, more research is required to fully understand the mechanisms of ISF-MBF interactions in the mosquito host. Reflecting on the limitations of the currently published studies, it becomes apparent that future research on ISF SIE should employ immunocompetent mosquito cell lines and validate in vitro findings in vivo. For in vivo work, the persistently infected mosquitoes should inherit ISF through vertical transmission (i.e., reared from infected eggs) and the secondary MBF should be delivered via blood feeding to recapitulate the natural infection process. In addition, it will be beneficial to use viral strains of ISFs and MBFs that are standardised across experiments when comparisons are made using the same virus. Such studies will help identify optimal ISF-MBF pairings that exhibit strong and consistent SIE in vivo. Current data indicates that the mechanisms underlying SIE by ISFs are likely multifactorial and unlikely to be mediated by siRNAs. Instead, they may involve depletion of host factors and competition for replication resources. Nevertheless, a deeper understanding of these mechanisms underlying SIE will be essential for developing a novel ISF-based biocontrol strategy to mitigate the transmission of MBFs.

## Data Availability

No new data were created or analysed in this study. Data sharing is not applicable to this article.

## Supplementary Materials

The following supporting information can be downloaded at: https://www.mdpi.com/article/10.3390/v18010115/s1, Table S1. Expanded summary table of homologous superinfection exclusion studies in orthoflaviviruses.
